# Gastric Cancer: How Can We Reduce the Incidence of this Disease?

**DOI:** 10.1007/s11894-016-0506-0

**Published:** 2016-05-16

**Authors:** Caroline M. den Hoed, Ernst J. Kuipers

**Affiliations:** Department of Gastroenterology and Hepatology, Erasmus MC University Medical Center, Rotterdam, The Netherlands

**Keywords:** Gastric cancer, Epidemiology, Prevention, *Helicobacter pylori*, Diet, Screening

## Abstract

Gastric cancer remains a prevalent disease worldwide with a poor prognosis. *Helicobacter pylori* plays a major role in gastric carcinogenesis. *H. pylori* colonization leads to chronic gastritis, which predisposes to atrophic gastritis, intestinal metaplasia, dysplasia, and eventually gastric cancer. Screening, treatment, and prevention of *H. pylori* colonization can reduce the incidence of gastric cancer. Other interventions that may yield a similar effect, although of smaller magnitude, include promotion of a healthy lifestyle including dietary measures, non-smoking, low alcohol intake, and sufficient physical activity. This chapter reviews interventions that can lead to a decline in gastric cancer incidence in high and low incidence countries.

## Introduction

**Fig. 1:**
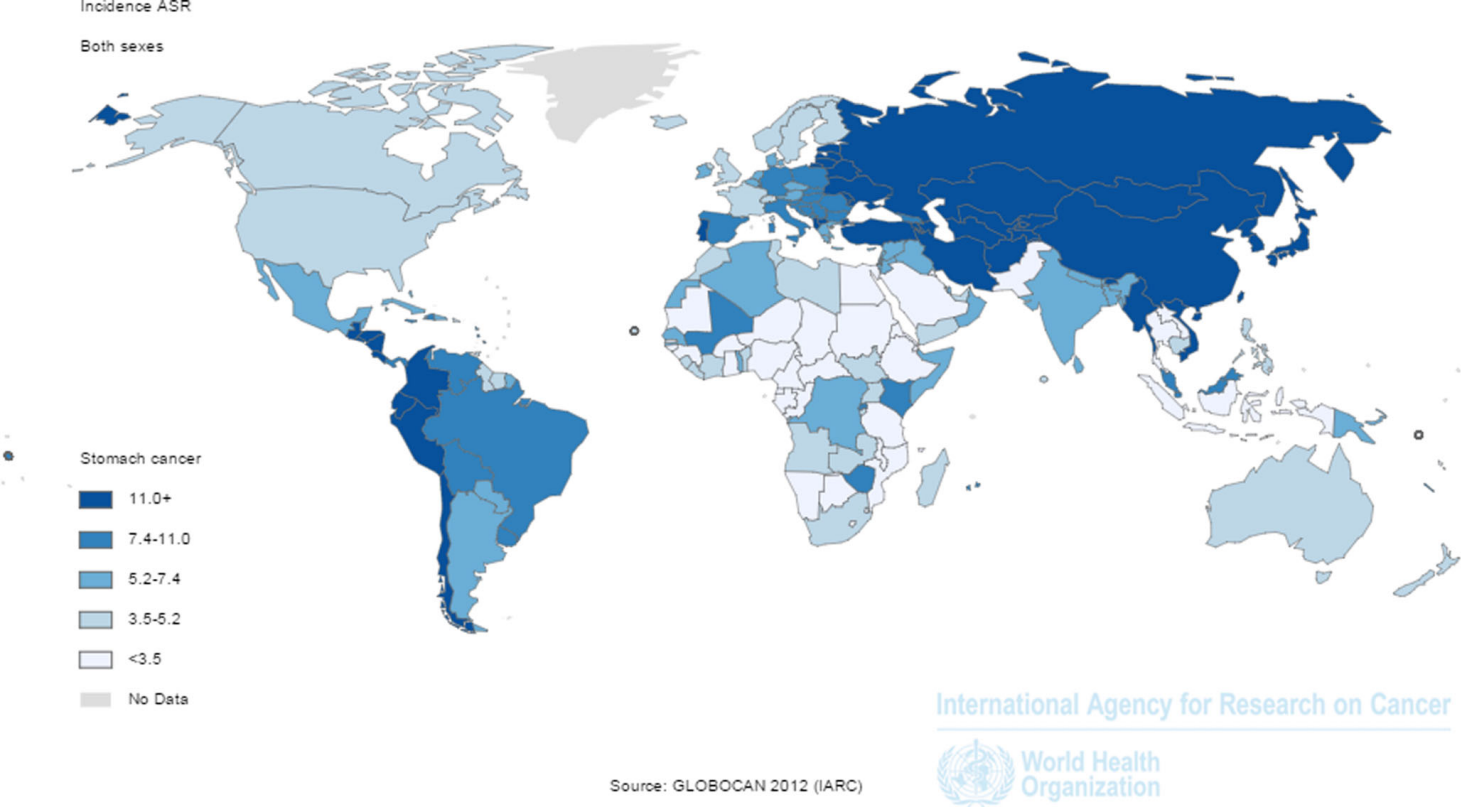
Incidence of gastric cancer in age-specific rate (ASR) worldwide in 2012. Reprinted by permission from GLOBOCAN 2012 (IARC), http://globocan.iarc.fr/, © IARC 2016, © WHO 2016

Despite its declining incidence, gastric cancer is still the fourth most common malignancy and remains the third leading cause of cancer-related death, following lung and liver cancer [1]. Despite medical progress, less than 30 % of subjects diagnosed with gastric cancer survive for more than 5 years. In 2012, almost a million gastric cancer cases were diagnosed and over 700,000 persons died from the disease [2]. The majority of cases occurred in Asia and Eastern Europe; however, gastric cancer is also common in certain areas of Middle and South America (Fig. 1). There are marked differences within many geographic areas, with considerably higher gastric cancer incidences among indigenous populations [3].

Most cases of gastric cancer are intestinal-type adenocarcinomas and the majority is localized in the antrum and body of the stomach. Non-cardia intestinal adenocarcinomas are thought to develop via a specific pathway first described by Correa in 1975 [4]. This pathway starts with chronic active gastritis leading to gland loss and development of atrophic gastritis, intestinal metaplasia, and dysplasia to eventually result in gastric adenocarcinoma.

The predominant risk factor for gastric cancer is *Helicobacter pylori* gastritis. The decrease in prevalence of *H. pylori* significantly contributed to the declining incidence of gastric cancer in many parts of the world. Nevertheless, the annual numbers of patients diagnosed with and dying from gastric cancer remained to increase till now, in particular due to the growing population in areas with high *H. pylori* prevalence [5]. Although the absolute numbers of gastric cancer are decreasing in Western countries, some studies suggest different patterns in various age groups, with in particular a significant increase in premalignant lesions and gastric cancer among younger patients [6, 7].

Colonization with *H. pylori* is not the sole determinant for the development of gastric cancer. Risk modulators are in particular related to lifestyle. The recognition of these risk modulators determines the options for prevention and intervention to decrease the incidence of gastric cancer.

## Helicobacter pylori

*H. pylori* gastritis primarily determines the risk for gastric cancer. Ubiquitous eradication of *H. pylori* by means of population screening and intervention programs would consequently lead to a major reduction in gastric cancer incidence worldwide. Large population-based studies in China and Taiwan, and case-control studies in Japan have shown a clear risk reduction by means of *H. pylori* eradication.

In 1995, the Shandong Intervention trial was initiated in Linqu, China. The goal of this intervention trial was to study the impact of screening and treatment for *H. pylori* on the incidence of precancerous gastric lesions and gastric cancer [8]. In total, 3365 subjects were randomly assigned to receive *H. pylori* eradication treatment or placebo. After 15 years of follow-up, *H. pylori* eradication was shown to significantly reduce gastric cancer incidence with 39 % compared to placebo treatment. Adjusting for other risk factors such as age, sex, alcohol, and smoking did not change the outcomes (OR 0.61 95 % CI 0.38–0.96) [9•]. Another study with approximately the same design was performed in Southern China [10]. The investigators randomized1630 *H. pylori*-positive subjects to receive either eradication treatment or placebo. After a follow up of 7.5 years, the incidence of gastric cancer did not significantly differ between the two groups. However, subgroup analysis of those subjects who had not had any signs of premalignant lesions at baseline showed that *H. pylori* eradication in this subgroup completely prevented gastric cancer whereas placebo treatment did not (*p* = 0.02) [10].

A population-based study in Matsu, a Taiwanese island with very high gastric cancer incidence, introduced an *H. pylori* test-and-treat strategy. The incidence of gastric cancer was compared to an earlier cohort with known *H. pylori* status but without eradication treatment for *H. pylori*-positives [11•]. This comparison suggested that *H. pylori* eradication led to a 25 % decline in gastric cancer incidence.

Several studies from Japan support the findings of these population studies. A study in Japanese peptic ulcer patients demonstrated that successful *H. pylori* eradication resulted in a decline in cumulative 5-year gastric cancer incidence from 3.8 to 1.2 % [12]. Another Japanese study identified 1476 *H. pylori*-positive patients, of whom 853 chose to receive eradication treatment and 624 did not. All patients were offered follow up. Eventually, 404 patients in whom *H. pylori* had been successfully eradicated and 304 patient who did not receive eradication treatment were followed up for 3 years. Gastric cancer occurred in 1.5 % in the eradicated group versus 4.3 % in the non-eradicated group (*p* 0.019) [13].

A Cochrane meta-analysis further addressed the impact of *H. pylori* eradication on prevention of gastric neoplasia [14•]. It included 6 randomized controlled studies with 6497 subjects, of whom 3294 received *H. pylori* eradication treatment and 3203 placebo. *H. pylori* eradication was associated with an RR of 0.66 (95 % CI 0.46–0.95) for development of gastric cancer compared to placebo [14•]. The studies included in this analysis were all performed in Asian populations, studies in other populations are till now lacking [14•].

Based on these data, the Japanese Society for *Helicobacter* Research advocated *H. pylori* eradication for prevention of gastric cancer. In 2013, the Japanese government decided to subsidize population *H. pylori* test-and-treat strategies to prevent gastric cancer. This has led to a massive increase in the number of annual treatment prescriptions to more than a million treatments per year [15].

While the use of *H. pylori* eradication for prevention of gastric cancer is thus expanding, it remains a matter of debate whether this is of sole benefit to subjects without premalignant lesions or whether it also decreases the incidence of gastric cancer in patients with premalignant lesions and those who were treated for early gastric cancer. A further paper based on the Shandong Intervention trial described the effects of *H. pylori* eradication treatment in subjects with different stages of precancerous lesions [16]. A total of 2172 subjects were included, of whom 1285 had intestinal metaplasia or dysplasia at baseline. *H. pylori* eradication treatment resulted in a statistically significant reduction in incidence of gastric cancer (HR 0.56, 95 % CI 0.34–0.91) and a non-significant reduction in gastric cancer mortality (HR 0.63, 95 % CI 0.29–1.37) in patients with intestinal metaplasia or dysplasia at baseline. These findings suggested that *H. pylori* eradication treatment is effective for prevention of cancer in subjects with intestinal metaplasia and dysplasia [16]. Other studies, such as the previously described mass eradication trial in Matsu, in contrast demonstrated no risk regression in subjects with intestinal metaplasia or dysplasia [11•].

The effect of eradication of *H. pylori* in patients treated for early gastric cancer and the risk of metachronous gastric cancer remains controversial. Three Japanese studies demonstrated a 58 to 100 % significant reduction in metachronous gastric cancer after *H. pylori* eradication during a follow-up period of 2 to 5 years [17–19]. In contrast, a retrospective multicenter trial did not show a reduction in the incidence of metachronous gastric cancer after a mean follow up of 3 years [20•]. More recently two studies further analyzed the effect of *H. pylori* eradication on metachronous gastric cancer. A Korean study retrospectively analyzed subjects who had undergone endoscopic resection for early gastric cancer [21•]. The authors compared patients without *H. pylori* infection with those who had undergone successful eradication and patients with persistent *H. pylori* infection. The incidence of metachronous gastric cancer was 10.9 per 1000 person-years in the *H. pylori*-negative group, 14.7 in the eradicated group, and 29.7 in the group with persistent *H. pylori* infection (*p* < 0.01).[21•]. Another Korean study with a median follow up of 4.5 years yielded similar results [22]. These studies suggest that *H. pylori* eradication after treatment for early gastric cancer can result in a reduction in the incidence of metachronous gastric cancer.

Population screening and eradication of *H. pylori*, the so-called population test-and-treat strategy, may be a cost-effective approach. A review by our group demonstrated that this strategy may be cost-effective in countries with high as well as low prevalence of *H. pylori*. All studies included in the review reported that screening for *H. pylori* to prevent gastric cancer in the general population costs less than US$50,000 per life year gained, a commonly used definition for cost-effectiveness in literature [23•].

Such an approach however has a number of potential limitations. These include the requirement for substantial population participation, treatment failure due to limited compliance and antimicrobial resistance, and reinfection. Although *H. pylori* reinfection rates tend to be low (i.e., less than 2 %), higher reinfection rates have been reported in countries such as Bangladesh and India with a high prevalence of Helicobacter in combination with poor sanitation and low standards of living [24]. Therefore, vaccination would theoretically be a perfect intervention; however, the development of a preventive and/or therapeutic vaccine has been disappointing until now [25].

## Screening and Surveillance of Premalignant Lesions

Since gastric cancer has specific precursor lesions, screening for identification of patients with these lesions and their surveillance may in theory also result in a lower incidence and mortality of gastric cancer. Population screening is most relevant for high incidence countries. It is in particular applied in Japan and Korea. Screening can be performed by endoscopy or serology. Endoscopic screening is based on thorough examination of the stomach lining either by endoscopic evaluation alone, as is the practice in Japan, with use of scoring systems [26•, 27]. Western clinicians generally prefer endoscopic examination in combination with random and targeted biopsy sampling with the use of the OLGA or OLGIM scoring systems as proposed in the MAPS guidelines [28, 29•, 30].

Serologic screening is in particular based on pepsinogen I, II, and their ratio, sometimes combined with *H. pylori* serology and measurement of fasting gastrin. Measurement of pepsinogen I and II offer a sensitivity of 69–70 % and a specificity of 88–97 % for the diagnosis of extensive chronic atrophic gastritis (CAG).[31•, 32] Subjects with pathologic serology are recommended to undergo endoscopy to assess the severity and extent of the (pre)malignant lesions.

The impact of endoscopic and serologic screening has both been studied in different populations. These studies firstly suggested that endoscopic screening in high prevalence countries leads to a reduction in gastric cancer deaths. A gastric cancer screening program in Niigata offered yearly screening with endoscopy [33•]. This resulted among participants in a 57 % reduction in gastric cancer deaths over a period of 5 years but the participation rate was only 25 % [33•]. A further case-control study by the same group evaluated the impact of endoscopic screening in five Japanese cities [34]. Participation in the screening program resulted in a 30 % reduction in gastric cancer mortality over a period of 3 years, but only 10 % of the target population participated. Since screening programs can only be effective on a population level if substantially higher participation rates are achieved, large efforts would be needed to make endoscopic screening a general success.

Serologic screening is potentially an elegant and more widely applicable alternative for endoscopic screening. Several studies based on a cohort of middle-aged Japanese employees demonstrated that the combination of *H. pylori* serology and serum pepsinogen levels can be applied as a screening method to identify subjects at high risk for gastric cancer [35, 36, 37•]. In total, 4655 employees were included and followed for a mean 16 years. The subjects were divided in four groups depending on *H. pylori* status and pepsinogen levels. The first group was *H. pylori*-negative with normal pepsinogen levels, the second group was *H. pylori*-positive with normal pepsinogens, the third group was *H. pylori*-positive with low pepsinogens, and the final group was *H. pylori*-negative with low pepsinogens. In comparison with *H. pylori*-negative subjects with normal pepsinogens, gastric cancer risk rose stepwise in *H. pylori*-positive subjects with normal pepsinogens (HR 8.9, 95 % CI 2.7–54.7), in *H. pylori*-positive subjects with low pepsinogens (HR 17.7, 95 % CI 5.4–108.6), and in *H. pylori*-negative subjects with low pepsinogens (HR 69.7, 95 % CI 13.6–502.9) [37•]. The latter group presumably has the most advanced atrophy and as a result has spontaneously eradicated *H. pylori*. A recent study conducted in Siberia confirmed these findings and demonstrated that the use of the pepsinogen I/II ratio was a reliable marker to identify patients at risk of developing gastric cancer, which could then be included in endoscopic surveillance [38]. There are some concerns on the limited predictive value of pepsinogens, and uncertainties on optimal cut-offs, but the negative predictive value is high [39]. A limitation of these tests is that use of a PPI may affect pepsinogens and gastrin levels.

A recent paper addressed the cost-effectiveness of gastric cancer prevention using a mathematical microsimulation model [40]. This model was based on the US population and focused on prevention by means of an *H. pylori* test-and-treat or detection and surveillance of premalignant lesions by means of pepsinogen measurements or endoscopy. These strategies reduced the lifetime risk for gastric cancer with respectively 0.2, 26, and 21 %. None of the interventions proved to be cost-effective except in subgroups at high risk.

Although endoscopic screening is cost-effective in low incidence countries, surveillance of patients with precancerous lesions may be. Surveillance would aim for early detection of cancer allowing for curative therapy and reduction of mortality. Recently, a Markov model-based study demonstrated that endoscopic screening of patients with extensive atrophic gastritis or intestinal metaplasia is cost-effective [41]. The 2012 MAPS guidelines propose endoscopic surveillance every 3 years for patients with extensive intestinal metaplasia [29•]. A Korean study based on 2845 subjects demonstrated that surveillance with a 3-year interval allowed early diagnosis of gastric cancer with similar benefits as annual or biennial surveillance. Prolonging the surveillance interval to 4 years or more however increased the risk of more advanced stage gastric cancer is [42•]. Studies on surveillance of patients with precancerous lesions in low incidence countries are rare but also suggest risk reduction and improved survival of gastric cancer [43].

## Healthy Lifestyle

Lifestyle factors such as diet, smoking, and use of alcohol significantly influence the risk of gastric cancer and are thus potential targets for disease prevention. This has been addressed in the prospective EPIC cohort study that aims to investigate the relationship between chronic diseases and lifestyle, diet, and environmental factors. The study included over 500,000 subjects in 23 centers across 10 European countries [44]. It showed that a healthy diet, no smoking or alcohol use, a normal BMI, and regular physical activity resulted in a 10 % reduction of gastric cancer risk.[45•]

### Diet

Several dietary factors modulate the risk for gastric cancer. This firstly pertains to a high intake of salt. A prospective study of 2476 subjects followed for a mean of 14 years demonstrated a significant association between high salt intake and gastric cancer with a hazard ratio of 2.87 (95 % CI 1.14–7.24) in *H. pylori*-positive subjects with atrophic gastritis [46]. An earlier population-based study obtaining sodium urine samples in populations of 24 countries demonstrated a significant correlation between stomach cancer mortality and the amount of sodium intake [47].

The EPIC study demonstrated an inverse correlation between the level of intake of fruit and vegetables and the risk of developing diffuse gastric cancer. However, this association seemed to be restricted to subjects who smoked [48•]. A further paper showed no association between the variety in vegetable and fruit intake and the risk of gastric cancer in low incidence countries [49•]. Similarly, no association was demonstrated between gastric cancer risk and the so-called Mediterranean diet [45•].

A further dietary factor that has been correlated to the risk of gastric cancer is fiber intake. Dietary fibers may neutralize potentially carcinogenic nitrites and thus reduce their intragastric concentration [50]. A meta-analysis showed a significant inverse correlation between dietary fiber intake and gastric cancer risk [51]. An increase of 10 g of dietary fiber per day was associated with a 44 % decreased risk of gastric cancer.

The role of vitamin intake in risk reduction of gastric cancer was studied in a recent meta-analysis. This provided evidence that even a relatively low dose intake of vitamin A (1.5 mg/day), C (100 mg/day), and E (10 mg/day) are associated with a significant decrease of gastric cancer risk, with a relative risk of 0.71 for vitamin A (95 % CI 0.62–0.810), 0.74 for vitamin C (95 % CI 0.69–0.79), and 0.76 for vitamin E (95 % CI 0.67–0.85) [52].

The Shandong intervention trial also studied dietary interventions to reduce gastric cancer incidence. Next to *H. pylori* eradication subjects were supplemented with garlic extract and garlic oil or with a mixture of vitamin C, E, and selenium. Vitamin treatment showed a trend toward a reduction of gastric cancer mortality which was not statistically significant (HR 0.55, 95 % CI 0.29–1.03) and garlic treatment did not affect gastric cancer mortality [9•]. This contrasted with a recent meta-analysis which concluded that any level of garlic intake was associated with a reduced risk of gastric cancer (OR 0.77; 95 % CI 0.6–1.0) [53]. High garlic intake reduced the risk further (OR 0.49, 95 % CI 0.38–0.62). A large cohort study on the effects of low intake of folate, methionine, vitamin B6, and vitamin B12 demonstrated an increased risk of esophageal squamous cell carcinoma, but no effect on gastric cancer risk [54].

Green tea is known for its strong antioxidant activity and can therefore enhance the overall chemopreventive effect of intragastric antioxidants. In a systematic review of six cohort studies on green tea intake and gastric cancer incidence, a limited protective effect of green tea was observed in women who drank at least five cups of tea per day.[55] A Cochrane meta-analysis demonstrated conflicting results in the included studies, concluding that there was moderate to strong evidence that green tea did not reduce the risk of dying from gastric cancer [56].

### Smoking and Alcohol

Smoking is also an established risk factor for gastric cancer [57]. The EPIC study demonstrated during a mean follow up of 11.4 years that never smoking or quitting >10 years previously was associated with a decreased risk of developing gastric cancer (HR 0.64, 95 % CI 0.54–0.75). This was the basis for a further simulation model-based study considering serologic screening, with either *H. pylori* testing or serum pepsinogens, to reduce gastric cancer risk in smokers and non-smokers. It was demonstrated that serum pepsinogen screening was not warranted for the general population because of an incremental cost-effectiveness ratio (ICER) of more than US$100,000 per QALY. However, in the subgroup of smokers, the ICER was below 93,000 per QALY in all of the proposed scenarios [40]. Furthermore, a strong association was demonstrated between alcohol intake and gastric cancer (HR 0.74, CI 053–0.97) [45•]. This association, however, was only found in heavy consumers of alcohol [58]. Not only are smoking and drinking an established risk factor for the development of gastric cancer but both factors may also be associated with failure of *H. pylori* eradication [59].

### Obesity

The EPIC study found no association between BMI and gastric cancer risk [60]. Similar results were obtained in a range of other studies, as well as in a meta-analysis that included 24 prospective studies [61].

## Pharmacological Interventions

Several studies and two recent meta-analyses looked at the impact of certain drugs, in particular statins and non-steroidal antiinflammatory drugs on gastric cancer incidence [62•, 63]. Most studies included in the meta-analyses were population-based. The randomized trials that were included had not been set up to identify a possible association between statins and gastric cancer. However, both meta-analyses demonstrated statin use was associated with a reduced risk of gastric cancer. The risk reduction was modest, and the number needed to treat to prevent one gastric cancer case would be high.

Cox-2 is involved in *H. pylori*-associated gastric carcinogenesis [64]. Some cohort studies reported that NSAIDs, such as aspirin or celecoxib, intake reduced the incidence of gastric cancer. A meta-analysis of 14 studies with 5000 cases and 30,000 controls demonstrated a significant protective effect of aspirin use on non-cardia gastric cancer (OR 0.62, 95 % CI 0.55–0.69) [65]. This observation was confirmed by a recent Korean population-based study. This study based on the Korean National Health Insurance claim database included 3907 cases using aspirin 100 mg daily and 7808 matched controls [66•]. Regular aspirin was associated with a non-significant reduced risk of gastric cancer (HR 0.71, 95 % CI 0.47–1.08; *p* = 0.1); subjects using aspirin for 3 years or more had a significantly lower gastric cancer risk (HR 0.4; 95 % CI 0.16–0.98; *p* = 0.04). Every year of aspirin use resulted in a further reduction of the gastric cancer risk [66•]

A randomized placebo-controlled trial aimed to study the role of the selective Cox-2 inhibitor, celecoxib, with or without *H. pylori* eradication in a high risk gastric cancer population [67•]. Celecoxib use was associated with a significant 52 % regression in premalignant gastric lesions compared to placebo, a percentage comparable to the effect of *H. pylori* eradication (54 %). However, the combination of initial *H. pylori* eradication followed by celecoxib did not result in additional beneficial effects compared to *H. pylori* eradication alone.

## Conclusions

Gastric cancer remains a prevalent disease with a very poor prognosis. Most patients with gastric cancer die despite aggressive multimodality treatment. This can generally be explained by the fact that most gastric cancers are diagnosed in a late stage. Therefore, measures are warranted focusing on prevention and early diagnosis.

*H. pylori* gastritis is the predominant risk factor for developing gastric cancer and most can be gained from *H. pylori* eradication. Eradication could either be population-based or focusing on subjects at highest risk. In high-incidence countries, population-based eradication seems a worthwhile intervention. However, the success of such intervention depends entirely on participation rates and the possible consequences of widespread *H. pylori* eradication are currently incompletely understood. A more targeted approach can consist of treating only high-risk groups or, for example, subjects over a certain age. Recent data suggest that *H. pylori* eradication at any stage of gastritis has a beneficial effect on the risk of developing gastric cancer and results in a decrease in gastric cancer incidence.

Other measures to reduce gastric cancer incidence and mortality aim to identify patients with premalignant lesions either by endoscopic or serologic screening. Endoscopic screening has the highest accuracy for assessment of the gastric mucosa but is on a population level only feasible in high-risk populations given the costs and the number needed to screen. Serologic screening is an elegant alternative. Surveillance of subjects with premalignant lesions is currently advocated in high- as well as low-incidence countries, and available data seem to support this practice.

Further interventions to lower gastric cancer incidence aim at promotion of a healthy lifestyle with adequate fiber, garlic and vitamin intake, modest alcohol use, and no smoking. Pharmacological interventions are of interest, but the results so far are disappointing. Statin use leads to a modest risk reduction and consequently a high number of subjects need to be treated long-term to prevent a single case of gastric cancer. Cox-2 inhibitors do not lead to an additional beneficial effect after *H. pylori* eradication.

In the coming decade, the effect of large-scale *H. pylori* eradication will be further elucidated, since the Japanese government started subsidization of population *H. pylori* eradication. This may also provide further answers on the positive and negative effects of *H. pylori* eradication. With respect to serologic screening, more data are needed on optimal selection of cut-offs and screening intervals in different populations. With these data, maximal impact of serologic screening can be clarified. Endoscopic surveillance is already in place in several countries and the effects of this intervention will become clear before long. The coming years will likely also bring more data on lifestyle and pharmacological interventions. Together, these measures will bring options to reduce the very high worldwide incidence and mortality of gastric cancer.
